# Psychological distress, General health and Life satisfaction among children with Thalassemia and children undergoing Haemodialysis

**DOI:** 10.12669/pjms.40.10.9412

**Published:** 2024-11

**Authors:** Aansah Ramzan, Saima Ahmad

**Affiliations:** 1 Aansah Ramzan, Department of Applied Psychology, Lahore College for Women University, Lahore, Pakistan; 2 Saima Ahmad, Department of Applied Psychology, Lahore College for Women University, Lahore, Pakistan

**Keywords:** Psychological Distress, General Health, Life Satisfaction, Thalassemia, Hemodialysis

## Abstract

**Objective:**

To compare and find out the relationship between psychological distress, general health and life satisfaction among children with thalassemia and children undergoing dialysis.

**Method:**

The current study utilized cross sectional research design. A representative group of children with Thalassemia (n=60) and children undergoing dialysis (n=60) with ages ranging from 6-14 years were included in the sample. The sample was drawn from Children’s Hospital and Sundas Foundation from February 2023 to August 2023 through a purposive sampling technique.

**Results:**

Psychological distress significantly correlates negatively with general health (-.34**) and life satisfaction (-.44**) in Children with Thalassemia. A significant negative relationship is also observed between psychological distress and general health (-.43**) and life satisfaction (-.54**) in Children undergoing Hemodialysis. Children undergoing hemodialysis experience more psychological distress whereas children with thalassemia have better general health. However, no significant difference was found in life satisfaction. The findings of the present study will be helpful for healthcare professionals and clinical psychologists to develop strategies and training programs which can increase resilience among patients. Training the patients and caregivers regarding how to fight chronic illnesses will lead to an improvement in their quality of life.

**Conclusion:**

Psychological distress is higher in children undergoing hemodialysis and general health is also not good but the level of satisfaction is almost equal between the two groups. This is justified that chronic illnesses do not preclude children from leading happy and satisfying lives.

## INTRODUCTION

Depression and anxiety are the main mental health issues of children with chronic conditions such as cancer, renal disease and thalassemia.^1^ It is also observed that the disease’s complexity and chronic nature harm sufferers’ quality of life and health. Children with chronic illnesses have poorer overall health and life satisfaction.^2^

A serious health issue, thalassemia threatens 72% of the world’s 229 nations. It poses a threat to 5.2% of the world’s population, 7% of expectant mothers, and 1% of couples. Thalassemia carriage is 5% worldwide.^3^ Children with thalassemia, who cannot have normal daily activity due to the fear of any injury that may cause life-threatening bleeding, are even more susceptible to anxiety and stress.^4^ Children with thalassemia have low general health and they are less satisfied with their lives.^5^ A sample of children with thalassemia from Iran showed that general and emotional health was compromised in these children.^6^

A study conducted in Pakistan showed that children with Thalassemia are affected tremendously. Their quality and satisfaction with life are affected emotionally and physically. The findings also revealed that the majority of the children were depressed and anxious.^7^ Another study conducted in 2024 showed that children with thalassemia have poorer satisfaction and quality of life.^8^ Another study showed that children with thalassemia show comprised quality of life, affecting their satisfaction with life.^9^ With a global incidence of 13.4% and an annual mortality rate of approximately 1.2 million (approximately), CKD is a severe public health concern.^10^ Children undergoing hemodialysis have a higher burden of fatigue, sleep-related impairment, psychological distress, impaired global health, and poorer family relationships compared with the general pediatric population.^11^ Children undergoing hemodialysis had lower general health and quality of life than children with thalassemia.^12^ A study conducted in 2024 showed that patients undergoing hemodialysis suffer from anxiety, depression and stress.^13^ Another study from Pakistan revealed that patients undergoing hemodialysis have low satisfaction with life.^14^ Patients undergoing hemodialysis suffer from anxiety, depression and poor quality of life.^15^ Surprisingly, children undergoing hemodialysis had higher levels of despair, and anxiety than children with thalassemia.^16^

Therefore, to document the difference between depression, anxiety and low satisfaction with life this study was conducted. The lack of research comparing children with thalassemia and children undergoing hemodialysis encouraged the researcher to explore the well-being and psychological aspects. This study aimed to assess levels of psychological distress, general health, and life satisfaction among children with different chronic conditions and compare them.

### Hypotheses:

There will be a negative relationship between psychological distress, general health and life satisfaction in both groups. Levels of psychological distress, general health and life satisfaction will be higher in children with Thalassemia than in children undergoing Hemodialysis.

## METHODS

The current study used cross-sectional research design. Research data for the current study was collected from Children’s Hospital and Sundas Foundation from April 2023 to August 2023. Permission was granted from the authors of the scales before the start of data collection. The researcher obtained permission from the heads of concerned departments of hospitals for data collection. The complete research protocol was administered to the participants including a consent form, demographics sheet and other scales. After data collection, the researcher thanked the participants with the assurance of confidentiality.

### Sampling Size:

A sample of children with thalassemia (n=60) and children undergoing hemodialysis (n=60) was included. The sample size was determined using G-power software.

### Ethical Approval:

Study was approved by the Ethics and Research Committee and Board of Studies of Lahore College for Women University (Reference no. 1571, dated: April 6, 2023) current study was conducted.

### Inclusion Criterion:

- Children with Thalassemia and children undergoing hemodialysis.
- Registered themselves at hospitals for the treatment of chronic disease.
- Patients with age range of 6-14 years of age.
- Informed Consent.

### Exclusion Criteria:

- Having any history of psychiatric problems (Depression, anxiety, and psychiatric disorders before the diagnosis of Thalassemia and dialysis).

### Instruments:

### Child Anxiety and Depression Scal^17^:

This scale is used to measure psychological distress. It’s a 47-item self-report questionnaire for children and adolescents that includes five subscales. Items are graded on a four-point scale ranging from 0 (“never”) to 3 (“always”). Chronbach’s alpha coefficient is .95. Higher score shows more psychological distress.

### Pediatric Global Health Scale^18^:

This scale is used to measure general health. It consists of seven questions, each with five response alternatives that address children’s general, mental, physical, and social health. Chronbach’s alpha coefficient is 0.88. A high score on this scale shows better health.

**Table-I:** Demographic characteristics of Children with Thalassemia (n=60) and Children undergoing Hemodialysis (n=60).

	Children with Thalassemia		Children undergoing Hemodialysis	

	F	%	F	%
***Age***				
6-8	22	37	22	37
9-11	27	45	23	38
12-14	11	18	15	25
***Gender***				
Male	30	50	30	50
Female	30	50	30	50
***Duration of disease in years***				
6-8	31	52	38	63
9-11	22	37	11	18
12-14	7	12	11	18

**Table II:** Correlation between Psychological Distress and General Health and Life Satisfaction in Children with Thalassemia (n=60).

Variables	1	2	3
Psychological Distress	-	-.34**	-.44**
General Health	-	-	.62**
Life Satisfaction	-	-	-

***Note:***

** p < .01.

**Table III:** Correlation between Psychological Distress, General Health and Life Satisfaction in Children undergoing Hemodialysis (n=60).

Variables	1	2	3
Psychological Distress	-	-.43**	-.54**
General Health	-	-	.59**
Life Satisfaction	-	-	-

***Note.***

** p < .01.

### Life Satisfaction Scale^19^:

This scale assesses a person’s overall assessment and quality of life based on five items scored on a five-point Likert scale ranging from “Strongly Disagree” to “Strongly Agree”. The scale has strong internal consistency of 86 higher the score higher the life satisfaction.

### Statistical analysis:

Hypotheses were tested using SPSS version 22.

## RESULTS

Correlation analysis shows significant negative relationship among all study variables. Psychological distress shows a significant negative correlation with general health (-.34**) and with life satisfaction (-.44**) in Children with Thalassemia. A significant negative relationship is also observed between psychological distress and general health (-.43**) and life satisfaction (-.54**) in Children undergoing Hemodialysis.

An independent sample t-test was carried out to compare the scores of each variable for children with thalassemia and children undergoing hemodialysis. Results reveal that children with thalassemia scored lower than children undergoing hemodialysis on psychological distress (t =3.52, p =.000). It means children undergoing hemodialysis experience more anxiety and depression as compared to other groups. Results also show that children with Thalassemia scored higher than children undergoing hemodialysis on general health (t =2.82, p=.005). Therefore, it was found that children with thalassemia have better general health as compared to children undergoing hemodialysis. However, no significant difference was found in life satisfaction.

## DISCUSSION

Patients with chronic conditions are very prone to psychosocial health problems therefore assessment of the mental health of this group is very essential. The most vulnerable group that is affected by these chronic conditions is children. The mental health of children not only affects them but the whole family. The current study included two groups with different chronic conditions.

Findings of the current study reveal that there is a significant negative correlation between psychological distress, general health and life satisfaction in children with thalassemia and children undergoing hemodialysis. The results are consistent with the outcomes of the previously conducted studies, which show that Thalassemia has the greatest impact on the emotional and social well-being of affected children.^20^ Literature also suggests that children with CKD reveal a higher burden of fatigue, sleep-related impairment, psychological distress, and impaired global health.^21^ Literature also shows that children with chronic diseases have psychological problems such as anxiety and depression and they have compromised health and life satisfaction in comparison with the general population.^22^ Patients receiving hemodialysis experience strong emotional difficulties. They experience anxiety, depression and low satisfaction with life.^23^ The findings of another study add that patients undergoing hemodialysis experience psychological distress and compromised health.^24^ Children with Thalassemia show lower physical functioning than emotional and school functioning.^25^

**Table-IV:** Independent sample t-test for comparison of means on all study variables.

Variables	Children with Thalassemia n=60		Children undergoing Hemodialysis n=60		t	p	Cohen’s d

	M	SD	M	SD			
Psychological Distress	29.49	7.77	32.26	5.74	3.52	.000	.41
General Health	64.54	8.15	61.76	8.82	2.82	.005	.33
Life Satisfaction	17.45	4.39	17.13	4.75	49	.655	.06

It was hypothesized that the level of psychological distress, and general and life satisfaction will be higher in children with thalassemia than in children undergoing hemodialysis. Previous literature shows that depression and anxiety are most prevalent in children undergoing hemodialysis.^10^ The general health of children with thalassemia is better than children undergoing hemodialysis.^20^ It shows that children undergoing hemodialysis have the lowest scores and suffer from poor general health compared to children with thalassemia. Children with CKD experience impaired quality of life in the physical and psychosocial functioning domains. A minor mean difference exists between life satisfaction in both groups. Previous literature suggests that children with chronic illness have lower general health and life satisfaction which preclude children from leading happy and satisfying lives.^26^

### Limitations:

The cross-sectional study design limited the researcher’s ability to uncover causal relationships between health-related quality of life and other factors. Another limitation of this study was that the researcher did not include all other psychosocial stressors or investigate potential biological factors (e.g., hormone levels, etc.).

## CONCLUSION

Psychological distress is higher in children undergoing hemodialysis. Children with thalassemia have better general health in comparison to children undergoing hemodialysis. So, to improve the general health of these children, appropriate programs should be planned and implemented to support them physically, mentally and socially. Furthermore, it is recommended that qualitative research should be initiated with these groups as they need more space to express their feelings.

### Recommendations

This research gives us a chance to further carry out more in-depth work on chronic diseases in pediatric samples. It is further suggested that more comparative studies should be conducted with different variables so that better management plans can be made and implemented according to the requirements.

### Author’s contribution.

**S A:** Concept, design, Data Analysis, Final Approval.

**A R:** Data Collection, Data entry, Write-up, and results interpretation.

All authors have read and approved the final version. They are also responsible for the integrity of the study.
